# Deep learning-based hemorrhage detection for diabetic retinopathy screening

**DOI:** 10.1038/s41598-023-28680-3

**Published:** 2023-01-27

**Authors:** Tamoor Aziz, Chalie Charoenlarpnopparut, Srijidtra Mahapakulchai

**Affiliations:** 1 Sirindhorn International Institute of Technology, Thammasat University, Bangkok, Thailand; 2 Kasetsart University, Bangkok, Thailand

**Keywords:** Computational models, Image processing, Machine learning

## Abstract

Diabetic retinopathy is a retinal compilation that causes visual impairment. Hemorrhage is one of the pathological symptoms of diabetic retinopathy that emerges during disease development. Therefore, hemorrhage detection reveals the presence of diabetic retinopathy in the early phase. Diagnosing the disease in its initial stage is crucial to adopt proper treatment so the repercussions can be prevented. The automatic deep learning-based hemorrhage detection method is proposed that can be used as the second interpreter for ophthalmologists to reduce the time and complexity of conventional screening methods. The quality of the images was enhanced, and the prospective hemorrhage locations were estimated in the preprocessing stage. Modified gamma correction adaptively illuminates fundus images by using gradient information to address the nonuniform brightness levels of images. The algorithm estimated the locations of potential candidates by using a Gaussian match filter, entropy thresholding, and mathematical morphology. The required objects were segmented using the regional diversity at estimated locations. The novel hemorrhage network is propounded for hemorrhage classification and compared with the renowned deep models. Two datasets benchmarked the model’s performance using sensitivity, specificity, precision, and accuracy metrics. Despite being the shallowest network, the proposed network marked competitive results than LeNet-5, AlexNet, ResNet50, and VGG-16. The hemorrhage network was assessed using training time and classification accuracy through synthetic experimentation. Results showed promising accuracy in the classification stage while significantly reducing training time. The research concluded that increasing deep network layers does not guarantee good results but rather increases training time. The suitable architecture of a deep model and its appropriate parameters are critical for obtaining excellent outcomes.

## Introduction

The International Diabetes Federation (IDF) estimated that 700 million people will be living with Diabetes mellitus (DM) by 2045^1^. DM develops fat and cholesterol in blood vessels that obstruct the flow of blood and nutrients required by human organs. The physiologic autoregulatory response to this progression increases the intracranial pressure of blood vessels^2^—This change in the retina yields rupture of small arteries that compiles Diabetic retinopathy (DR). DR is a retinal compilation that damages blood vessels, and DM is one of the causes of its development. DR cannot be cured permanently, but its progression rate can be reduced significantly by effective control of serum glucose, blood pressure and timely treatment.

**Figure 1:**
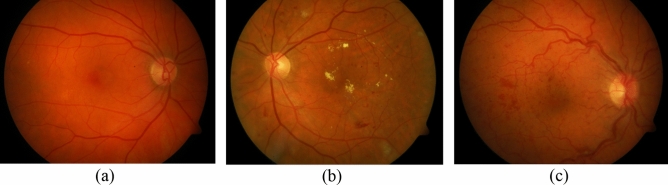
Stages of diabetic retinopathy in fundus images (**a**) Normal image (**b**) NPDR (**c**) PDR.

DR is broadly categorized into non-proliferative diabetic retinopathy (NPDR) and proliferative diabetic retinopathy (PDR). NPDR is classified by visible pathological features like microaneurysms (MAs), hemorrhages (HEs), exudates, and microvascular abnormalities. In comparison, PDR is diagnosed by the emergence of new blood vessels called neovascularization^3^. Figure 1 depicts two stages of DR.

MAs and HEs are grouped into red lesions. MAs are the earliest clinically manifest of DR and appear as small circular red dots in fundus images. Causes of MAs development are endothelial cell dysfunction, hypercoagulability, and atherosclerosis^4^. When MAs are weakened enough to rupture and swell, they rise to HEs. These dot-HEs are indistinguishable from MAs. HEs are disastrous for eyesight, predominantly when emerging in the macular region. Therefore, they often lead to significant and perpetual ocular impairment^5^.

Fluorescein angiography (FA), optical coherence tomography (OCT), and ophthalmoscopy are eminent methods of DR diagnosis. FA is a standard gold method and is capable of assessing microvascular changes. It helps to determine the origin of the leakage. Conversely, FA is time-consuming, invasive, and requires injection of intravenous fluorescein dye that may cause adverse side effects^6^. OCT is advanced for microvascular evaluation and captures a cross-section of the retina. OCT is non-invasive and does not require fluorescent dye injection. OCT assists in determining the progression rate of compilation and efficacy of therapy^7^, but complexities and risks are evident. Therefore, it necessitates computationally efficient, affordable, and non-invasive diagnostic method. Retinal ophthalmoscopy can be an alternative to address these problems^8^. It allows examination of retinal structures and can identify clinical pathological symptoms. This method does not constitute any risks; therefore, it is used for regular eye examinations.

Fundus images are often blurry and poorly illuminated. Intensity profiles of HEs appear similar to the macula, blood vessels, and dark regions caused by the lighting conditions. Therefore, the classification of HEs from other objects is a challenging task. Furthermore, the lack of a standardized description of HEs locations obstructs comparing various prognosis approaches. Localization of hemorrhage is a critical subject when prescribing treatment by an ophthalmologist. For instance, macular hemorrhage is more disastrous than other HEs^9^ because it deteriorates central vision. Therefore, the detection of HEs with their precise locations is exceptionally imperative. A fundus image may have many HEs with different sizes, and the intensity profiles of their surrounding regions are inconsistent. Hence, it demands an adaptive segmentation process to deal with differing sizes and inconsistent regions for effective HEs identification. Effective implementation of deep models requires sufficient data for training a model. A deep network provides obscure results that are difficult to interpret when trained on insufficient training samples. Therefore, these challenges compel the development of an efficient computer-based technique for HEs recognition that may act as a second interpreter. It may assist medical experts in prescribing appropriate treatment due to the heterogeneous nature of treatment modalities.

In this study, the impediments mentioned above to HEs detection are incorporated. Modified adaptive gamma correction by employing gradient information adjusts the image’s brightness level for better contrast. The algorithm localizes prospective candidates with accurate locations using empirical image processing. This step eliminates redundant information and expedites the detection process by considering only those objects that are likely to be HEs. Besides, this localization process provides sufficient data by generating windows/patches for the training of the classification model. It helps to design a computer-aided design (CAD) that works efficiently for limited images. The HEs can be located anywhere with random sizes in the retinal region and surrounded by areas with different intensity profiles. Therefore, the novel smart window-based adaptive thresholding incorporates regional diversity and segments HEs regardless of their sizes and encompassing regions. Removing unrequired intensity information from the image makes the HEs classification task simple. Therefore, the shallow Hemorrhage net (HemNet) is designed to learn from deep features in the training stage and differentiates HEs from other retinal structures. Despite HemNet containing fewer deep layers, it is competitive with other deep models that are extensively deep. Its generalization capability is proven through synthetic experimentation, which shows that HemNet takes less training time and has higher evaluation metrics than other models.

The research work is organized as follows. Section one is the introduction and importance of the research problem. It illustrates the application of computer-aided design in ophthalmology, the impediments to detecting the HEs, and how difficulties are addressed to identify HEs automatically. Section two is the review of relevant methods proposed by the research community. Section three explains the propounded preprocessing stage for quality enhancement and discusses the estimation of prospective HEs candidates. Then the segmentation technique is explained thoroughly to generate sufficient windows/patches for training a novel HemNet. “Experimental results” section is about experimentation design and compares deep models using evaluation metrics. It also includes synthetic experimentation to validate the performance of HemNet. Section five is the conclusion and contains the points for future consideration.

## Related Work

Li et al.^10^ comprehensively review fundus photography’s deep learning applications. It has been reported that training convolution neural networks (CNN) is time-consuming. Khojasteh et al.^11^ use probability maps provided by the SoftMax layer for retinal abnormalities classification. Enhancement and segmentation are performed, and then annotated patches train CNN. A trained classifier analyzes testing data where the probability map identifies the DR symptoms. In this study, 18,882 HEs-related examples are utilized for network training, which requires a long time to train CNN. A fully automated HEs detection method is presented by Lehmiri and Shmuel^12^. Variational mode decomposition processes retinal fundus images to obtain high-frequency components. Four sets of texture features train a classifier that discriminates healthy from unhealthy images. Short processing time with higher accuracy is reported. Orlando et al.^13^ detected red lesions using hand-crafted and deep features. The augmented ensemble vector and random forest (RF) classifier identify red lesions in fundus images. Three RF classifiers are trained using ensemble vector, hand-crafted, and deep features, and their performances are compared. Son et al.^14^ develop a method for the simultaneous detection of retinal pathologies that include HEs. A large amount of data trains CNN, and results are compared with ophthalmologists’ manual annotations of retinal fundus images. CNN architecture provides probability and a heatmap, which is a normalized single-channel low-resolution image. This image contains lesions identified by the algorithm and is superimposed onto the original image to highlight lesions. Most importantly, the classification result correlates with the gray values of the heatmap to identify the locations. Gayathri et al.^15^ devised a novel CNN architecture to extract features from the retinal fundus images. Extracted deep features train various classifiers like support vector machine (SVM), AdaBoost, Naïve Bayes, RF, and J48. These classifiers are evaluated using specificity, precision, recall, false positive rate (FP), Kappa-score, and accuracy. The performance of the J48 classifier with the proposed feature extraction model is the best among all classifiers. Hacisoftaoglu et al.^16^ developed an automated diagnostic model specially designed for low-quality images captured using smartphones’ small field of view (FOV). The transfer learning approach employs well-known architectures of Alexnet, Googlenet, and Resnet50. These CNN models evaluate the effects of single, cross, and multiple datasets. The proposed Resnet50 model is applied to smartphone-based synthetic images, evaluated on an independent dataset, and yields promising results. Qureshi et al.^17^ propounded a label-efficient CNN called active deep learning (ADL-CNN) using expected gradient length (EGL). ADL-CNN selects critical samples by using ground truth labels for feature extraction. Then, retinal pathologies are segmented and graded according to severity levels. Hemanth et al.^18^ propounded a compound of image processing and a deep learning-based hybrid method for DR diagnosis. Image quality is enhanced using histogram equalizations. Each channel of a color image is enhanced and concatenated to improve quality. The sizes of images are normalized to 150 × 225 for the CNN classification stage. Four hundred retinal fundus images of the MESSIDOR dataset are used for validation, and the algorithm reports promising results.

Training CNN is time-consuming, and obtaining high accuracy is a challenging task. Some techniques increase classification metrics and reduce training time using various approaches. The first approach estimates required objects that are more likely to be objects of interest. The second approach includes and excludes training examples based on their contribution to classification. For instance, a two-stream red-lesions detection is proposed by Asiri et al.^19^. Regions of prospective candidates are extracted using vessel segmentation and morphological operations to reduce computational complexity. This preprocessing step yields better results because it explores prospective candidates, enhancing accuracy in the classification stage. Pre-trained visual geometry group (VGGNet) is tuned for vessel and potential candidates’ segmentation. These candidates are classified using Faster RCNN, which provides promising results. Grinsven^20^ proposed a technique for HEs detection to expedite the training process. Training examples are heuristically sampled and misclassified negative samples are dynamically selected. Performances of trained CNNs using selective sampling and without selective sampling are evaluated. The method reduces a substantial number of epochs and provides promising classification results.

The proposed detection scheme ventures similar approaches presented earlier to reduce training complexity. First, prospective HEs candidates are estimated to eliminate irrelevant objects. It reduces the number of examples that are to be explored by CNN and expedites the detection process. Secondly, windows of fundus images are segmented to remove unrelated information before being provided to a CNN model. This step reduces intensities from the windows, and CNN extracts the features related to the HEs. Therefore, this research evaluates various CNN models trained on data from a small dataset.

## Method

### Dataset description

**Figure 2:**
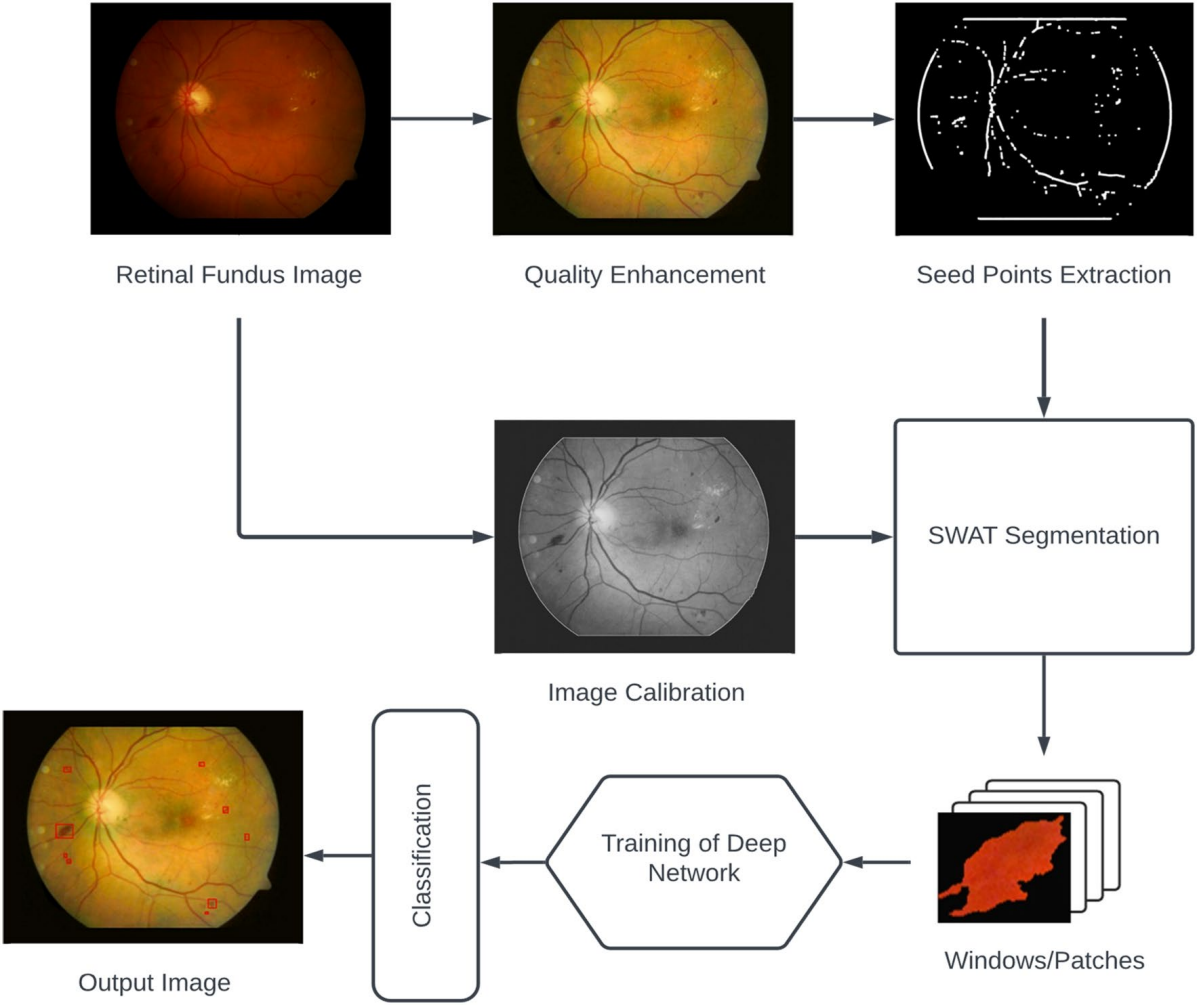
Illustration of the proposed detection technique.

Two datasets of DIARETDB1^21^ and DIARETDB0^22^ are employed for experimentation. The first dataset contains 89 fundus images, of which five are standard, and the rest have various retinal pathological symptoms. The second dataset contains 130 images, of which 110 have DR signs. These images are captured by the 50-degree field of view using a fundus camera under various illumination conditions. Figure 2 depicts multiple steps of the propounded detection scheme.

### Methodology

#### Quality enhancement

Qualities of retinal fundus images vary due to different illumination conditions. A rigorous visual inspection of images reveals that excess light provides over-saturation in some regions, the edges of required objects are blurry, and insufficient light produces dark regions. Therefore, the qualities of fundus images are enhanced to reduce the effects of low-quality characteristics of digital images. First, the contrast of an image is enhanced using contrast-limited adaptive histogram equalization (CLAHE)^23^.

An adaptive process is required to adjust brightness levels because some images have adequate brightness levels while some are dark. The brightness level is adjusted using gamma correction^24,25^, and the gradient value is used to produce the adaptivity. This process is applied to the individual color channel. Let $\varphi$ be a threshold value that separates smooth regions from the edges of the Sobel gradient image and is considered as the brightness interpretation of an image. Low correction is required when $\varphi$ is large, which yields an adequate brightness level of the image. Conversely, high correction applies when $\varphi$ is small, which reveals a low brightness level of the image. Therefore, $\gamma$ is calculated by adjusting $\varphi$ as:

1 $$\gamma =\alpha *\frac{\left\lfloor {\varphi \times 100} \right\rfloor}{10}$$

2 $$V\left(x,y\right)={I}_{\mathrm{max} }{\left(\frac{I}{{I}_{\mathrm{max} }}\right)}^{\gamma }$$

where $\alpha$ is the brightness adjustment coefficient and used as $\alpha$ = 2. Equation (2) adjusts the brightness level of the image, where ${I}_{ }\in \{0,{I}_{max}\}$ and ${I}_{\mathrm{max}}$ is the maximum intensity of an image.

Blurriness reduces texture and edge information in the image. The green component is sharpened using non-linear unsharp masking to improve edge information. This method computes the intensity difference using the fuzzy relationship between focused and neighboring pixels in a $3\times 3$ window. Pixels are sharpened using non-linear relationships depending on the luminance difference of the adjacent pixels^26^. The sample of the quality enhancement method is given in Fig. 2.

#### Seed points extraction

**Figure 3:**
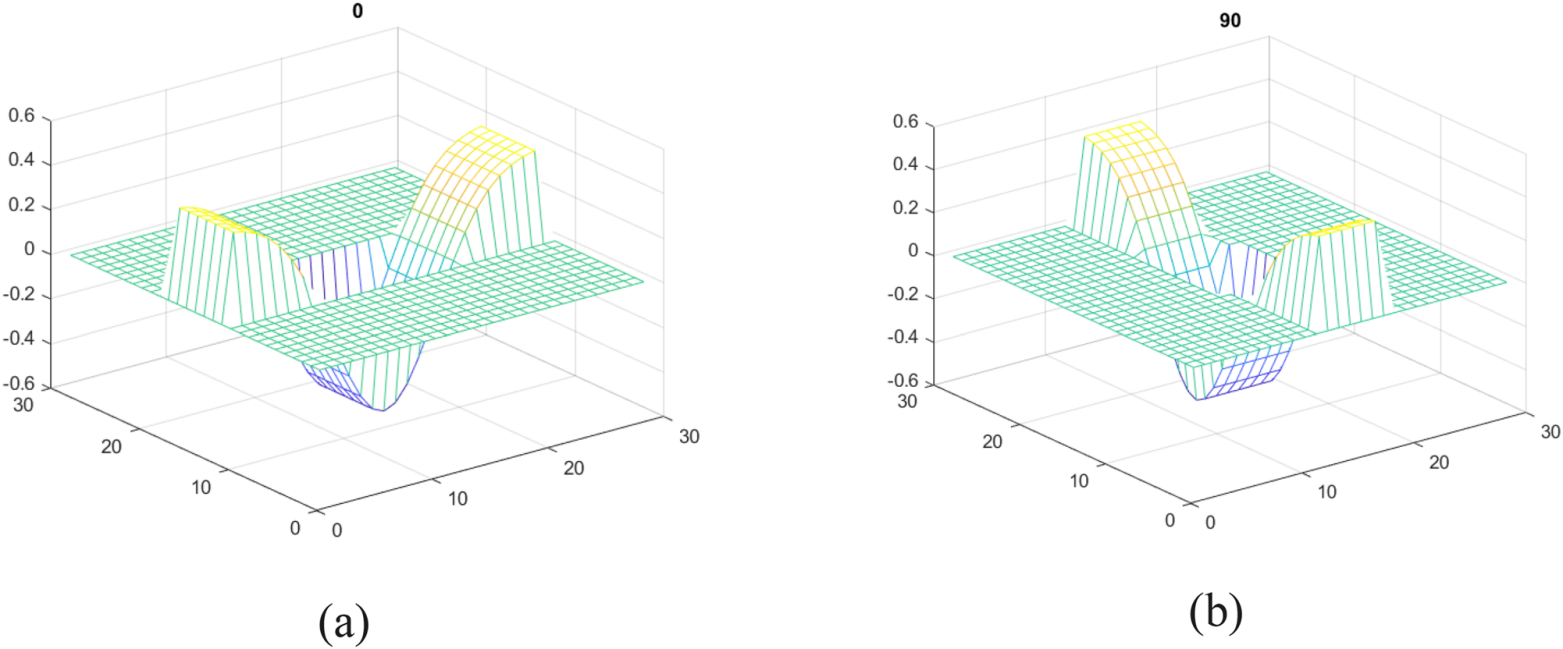
Rotation of matched filters (**a**) kernel with 0° angle (**b**) kernel with 90° angle.

The intended research work automatically detects HEs using seed points. Seed points highlight the locations of prospective HEs candidates. HEs share intensity information with blood vessels because of their similar appearances, and they are dark objects surrounded by bright regions. These characteristics lead to the development of an inverted Gaussian-based matched filter^27^. The kernels of the matched filter are applied to enhance dark objects, including HEs. Kernels with ${0}^{^\circ }$ and 90° angles can be depicted in Fig. 3.

The matched filter effectively enhances HEs and blood vessels due to the strong correlation. However, it provides a low response to the intensity variations of the image due to a weak correlation. GLCM-based local cross-entropy thresholding^28^ removes the low response of the matched filter. This method estimates the intensities into background to the background (BB), foreground to foreground (FF), background to the foreground (BF), and foreground to background (FB), given a threshold value $t$. BB and FF represent smooth regions, while BF and FB contain the information of edges. The edges do not contain substantial information; therefore, the entropies of BB and FF quadrants are used to find the optimum threshold value ${t}^{*}$, successively.

The formation of HEs starts from the leakage of blood vessels, so some of them can be attached to the blood vessels. Their isolation is critical for the early detection of DR. Mathematical morphology analyzes the spatial structures. The morphological opening is applied to break the larger objects than structuring elements. A square structure element of size $11 \times 11$ is used in our experiment. The image containing the seed points for subsequent segmentation is shown in Fig. 2.

#### Image calibration

HEs emerge at the retina’s vitreous humour, and the black background does not contain any information. Black background misleads the detection process for the HEs at the retina’s rim. Therefore, the illumination of the black background assists in automatic detection and reduces the search space. The median filter is applied on the green channel to suppress the random intensity variations on the black background then the image is binarized to get the retinal mask. The eroded mask is subtracted from the retinal mask to achieve the exact retinal boundary. The hand-crafted image is obtained by adding complimented retinal mask multiplied by the average gray value, retinal periphery, and the enhanced green channel. Figure 2 shows the calibrated image with a bright background where the retinal information is not undermined and is used for feature extraction.

#### SWAT segmentation

Segmentation of two types of HEs is challenging and requires a highly strenuous and intelligent method. First, the HEs that are located at the retinal periphery blended with the dark background. Secondly, those are attached to the blood vessels. The black background has been illuminated using image calibration that aids in performing segmentation at the retina’s border. Smart window-based adaptive thresholding (SWAT) can sense gray dissimilarity between HEs and blood vessels. It also provides adaptivity in segmentation to deal with the HEs encompassed by various regions. SWAT uses Otsu’s method for thresholding and finds the effectiveness value $\eta$ using:

3 $$\eta =\frac{{\sigma }_{B}^{2}\left({\tau }^{*}\right)}{{\sigma }_{T}^{2}}$$

where $\eta$ is the ratio of inter-region variance ${\sigma }_{B}^{2}({\tau }^{*})$ to the total variance ${\sigma }_{T}^{2}$ of the image. The value of ${\sigma }_{B}^{2}({\tau }^{*})$ depends upon the selection of an appropriate number of regions $\varrho$ in a window ${W}\left(x,y\right)$. SWAT finds the optimum number of regions $\varrho$, iteratively using Eq. (4), that yields maximum effectiveness value $\eta$ within the range 0–1 and produces robustness in the segmentation process. $\varrho$ generates a vector $\vartheta$ containing $\varrho -1$ threshold levels. The window is binarized using Eq. 5.

4 $$\vartheta =\left\{\begin{array}{*{20}l}\varrho \to \varrho +1, if \eta <0.8, AND \varrho \le 20\\ stop, otherwise\end{array}\right.$$

5 $${W}_{1}\left(x,y\right)=\left\{\begin{array}{*{20}l}0, if {W}\left(x,y\right)>min(\vartheta )\\ 1, else\end{array}\right.$$

HEs with bigger sizes are prioritized because they are more disastrous for eyesight than small HEs. Therefore, two more oversized objects are retained, and the rest are removed. This maneuver reduces the risk of false detection because dark shades are often bigger and may mislead the segmentation stage. Furthermore, the window originates from a seed point that probably belongs to a HE. This estimation criterion is proposed because seed points are extracted using the characteristics of HEs. Therefore, the object with a minimum distance is a HE, and the other one is removed by computing the Euclidean distance as:

6 $${d}_{i}=min\sqrt{{\left\{{W}_{1}\left({x}_{c}\right)-{I}_{i}\left(x\right)\right\}}^{2}+{\left\{{W}_{1}\left({y}_{c}\right)-{I}_{i}{\left(y\right)}\right\}}^{2}}$$

where ${I}_{i}\left(y\right)$ and ${I}_{i}(x)$ are the $y$ and $x$ spatial locations of the ${i}_{th}$ object, and $i=\{\mathrm{1,2}\}$. The object’s spatial locations assist in segmenting the complete HEs using SWAT. The size of the bounding box is increased to the particular directions according to the border pixels using the following relations:

7 $$V= \left\{\begin{array}{c}{v}_{1}\to {v}_{1}-5, if {q}_{1}=1 AND {v}_{1}\cap S\\ {v}_{2}\to {v}_{2}-5, if {q}_{2}=1 AND {v}_{2}\cap S\\ {v}_{3}\to {v}_{3}+10, if {q}_{3}=1 AND {v}_{3}\cap S\\ {v}_{4}\to {v}_{4}+10, if {q}_{4}=1 AND {v}_{4}\cap S\end{array}\right.$$

where $S$ is the search region to achieve automation in the detection process. The search region $S$ restricts SWAT from searching within the image domain. The region $S$ is obtained by extending the retinal mask eighty pixels wide in each direction. It provides sufficient space, especially for those HEs that reside at the retinal periphery. The vector $Q$ contains binary variables $q1,q2, q3,$ and $q4$ for corresponding left, top, right, and bottom border pixels. Equation 7 updates the vertices ${v}_{1}, {v}_{2}, {v}_{3},$ and ${v}_{4}$ of vector $V$, accordingly. The window sample in Fig. 2 is used to train a classifier.

#### Training of deep network

**Figure 4:**
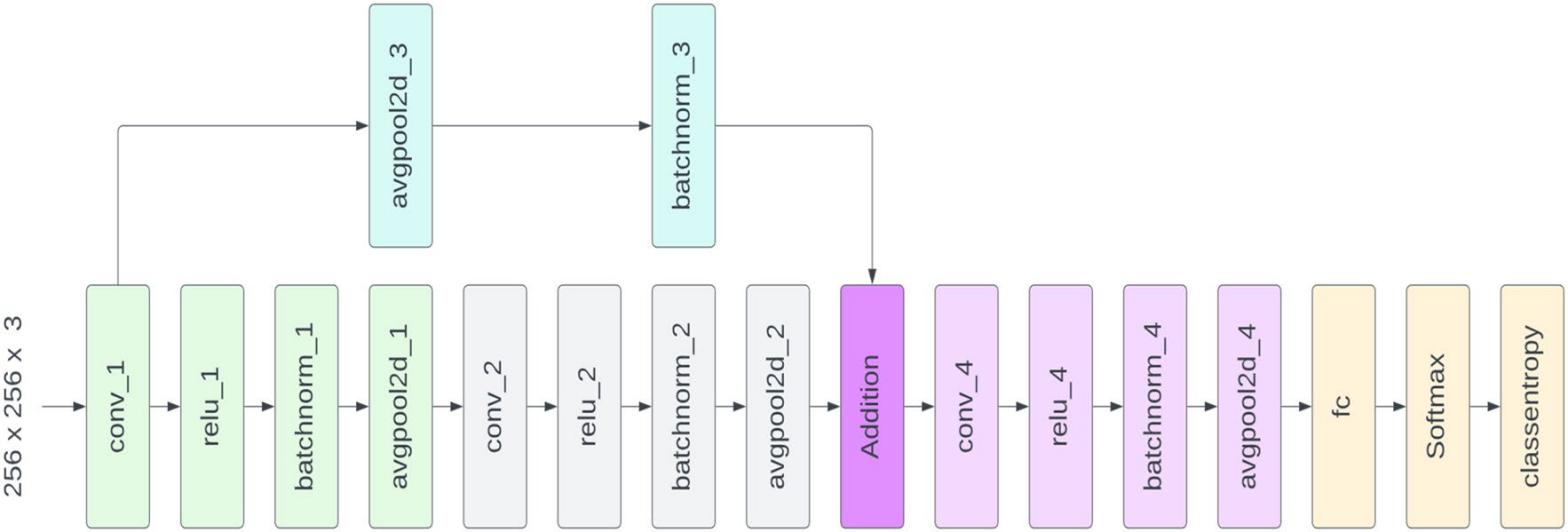
Overview of Proposed HemNet.

Some seed points belong to other retinal structures like blood vessels, dark shades, and intensity variations. SWAT segments and other retinal structures originated from those seed points and are redundant in our experiment. Therefore, it demands a classifier to discriminate HEs in the detection process. We propose a novel HE network (HemNet) that is shallower than conventional deep models and efficiently classifies HEs from other retinal structures. Our deep model contains nineteen individual layers, including input and output layers. Table 1 elaborates on the architecture of the proposed HemNet and can be depicted visually in Fig. 4.

**Table 1:** The architecture of the proposed deep convolution network.

Layer type	Layer name	Kernel size	Number of filters	Stride length	Output shape
Image input	–	–	–	–	(256, 256, 3)
Convolution	conv_1	$11\times 11$	16	3	(86, 86, 16)
ReLU	relu_1	–	–	–	–
Batch normalization	batchnorm_1	–	–	–	(86, 86, 16)
Average pooling	avgpool2d_1	$2\times 2$	–	–	(86, 86, 16)
Convolution	conv_2	$5\times 5$	16	2	(43, 43, 16)
ReLU	relu_2	–	–	–	–
Batch normalization	batchnorm_2	–	–	–	(43, 43, 16)
Average pooling	avgpool2d_2	$2\times 2$	–	–	(43, 43, 16)
Average pooling	avgpool2d_3	$5\times 5$	–	–	((43, 43, 16)
Batch normalization	batchnorm_3	–	–	–	(43, 43, 16)
Addition	Addition	–	–	–	(43, 43, 16)
Convolution	conv_4	$11\times 11$	16	2	(22, 22, 16)
ReLU	relu_4	–	–	–	(22, 22, 16)
Batch normalization	batchnorm_4	–	–	–	(22, 22, 16)
Average pooling	avgpool2d_4	$2\times 2$	–	–	(22, 22, 16)
Fully connected	Fc	–	–	–	(1, 1, 2)
SoftMax	SoftMax	–	–	–	(1, 1, 2)
Classification output	Classentropy	–	–	–	(1, 1, 2)

After segmenting the required objects using seed points, windows are extracted by the vector $V$ from an enhanced colored image. This colored data is labeled and transformed into HSV^29^ and CIE Lab^30^ color spaces inspired by the HEs detection using conventional features^31^. For instance, the edges of HEs are sharper than the edges of the macula, so the first and second-order based gradient features distinguish HEs from the macula. The blood vessels are line-shaped retinal structures, and HEs are comparatively rounded-shaped elements. So, connected component-based shape features classify them. Color features discriminate the objects of interest from black shades in the green channel. Therefore, similar characteristics are transformed to train HemNet that models conventional features. Three channels are used to train the network: the green channel of RGB, the value channel of HSV, and the luminance channel of CIE Lab color spaces. In addition, surrounded regions of HEs are eliminated, and the segmented objects are used in the training so the deep network can effectively classify HEs from blood vessels and other retinal structures.

## Experimental results

### Experimental composition

The machine with a 2.4 GHz Core-i5 processor, 16 GB RAM, and a single 6 GB GPU is used for the implementation. The performance of our detection method is evaluated using the DIARETDB1 and the DIARETDB0 datasets. Images of the DIARETDB1 dataset that contain HEs are used for the training. Our proposed algorithm intends to detect HEs, and the interpretation of HEs from ground truths reveals that forty-five out of eighty-nine images contain hemorrhages. These forty-five images are separated into training and testing sets. The training set comprises twenty-five images, and the testing set includes twenty images. The training set is further divided into the training and validation sets. These examples are annotated using the ground truths. Twenty images of the DIARETDB1 dataset are tested randomly, and the results are compared. Twenty images of the DIARETDB0 dataset are randomly taken to independently benchmark the algorithm’s performance . The classification results are determined using sensitivity (SE), specificity (SP), accuracy (AC), and precision (P). AC is an effective measurement for evaluating models’ performance because it is a ratio of classified examples from the total number of samples^32^. These evaluation metrics are measured as follows:

8 $$SE=\frac{TP}{TP+FN}$$

9 $$SP=\frac{TN}{TN+FP}$$

10 $$P=\frac{TP}{TP+FP}$$

11 $$AC=\frac{TP+TN}{TP+TN+FP+FN}$$

Time is a critical factor for screening and diagnosing diseases using medical images. Deep networks are efficient but often take a long time to train. Insufficient training of networks reduces classification accuracy. Therefore, the proposed network is analyzed using training time versus classification accuracy by synthetic experimentations using two datasets. Image of concrete crack^33^ and Modified National Institute of Standards and Technology (MNIST)^34^ digits datasets benchmark the HemNet. Thirty thousand images of the concrete crack dataset are included, where training, validation, and testing sets consist of twenty thousand, five thousand, and five thousand, respectively. Ten epochs are used with a learning rate of 0.009 in this trial. This experiment is assessed using training time, validation accuracy, and testing accuracy, and their statistics are provided in Table 4. Ten thousand images of the MNIST digits dataset are employed for the second synthetic experiment. One thousand images are equally distributed for validation and testing. While eight thousand images train the network using fifteen epochs and a 0.01 learning rate. The results of the digits classification are provided in Table 5.

### Analysis of results

**Figure 5:**
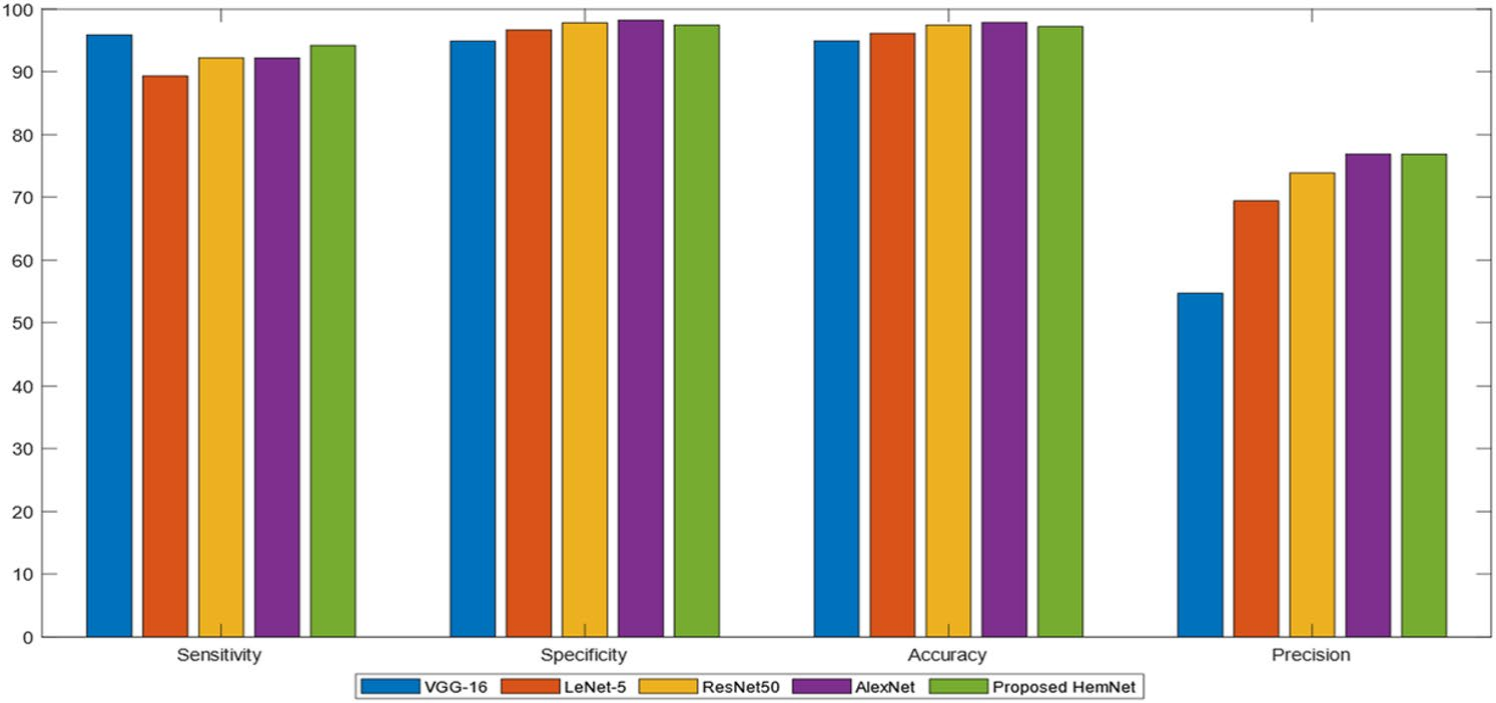
Comparison of deep models with proposed HemNet Network on DIARETDB1 Dataset.

The performance of HemNet was compared with the state-of-the-art CNN models for HE detection and classification. Despite the fact that HemNet was a shallower network, it provided competitive results when compared with other deep networks like LeNet-5^35^, AlexNet^36^, ResNet50^37^, and VGG-16^38^ on the DIARETDB1 dataset. The SE of the HemNet was closer to the SE of VGG16, which was the highest, stating the true positive detection rate of HEs. While HemNet marked 94.21% SE, which was higher than LeNet-5, ResNet50, and AlexNet. It shows that HemNet identifies most of HEs with low false-negative rate. Additionally, the 94.76% SP of HemNet was slightly less than AlexNet but greater than VGG-16 and LeNet-5. The difference in SP was negligible when comparing ResNet50 and the proposed model. SP shows the misclassification of some segmented objects as HEs but they do not belong to this class. AC of ResNet50 and AlexNet was 97.46% and 97.88% and were the highest, but HemNet scored 97.19%. This difference was negligible and not critical because the SE of the proposed HemNet is higher than ResNet50 and AlexNet. It shows truly classified HEs detection of the HemNet. The AC of HemNet was greater than VGG-16 and LeNet-5. AlexNet and the proposed model marked high *P* of 76.90% and 76.87%, respectively. These statistics validated the excellent performance of HemNet in terms of misclassification rates; false-positives and false-negatives. The classification results of various deep learning models on DIARETDB1 are statistically compared in Table 2 and visualized in Fig. 5.

**Table 2:** Classification of HEs using various models on the DIARETDB1 Dataset.

Methods	SE (%)	SP (%)	AC (%)	*P* (%)
VGG-16	95.88	94.87	94.93	54.69
LeNet-5	89.35	96.69	96.13	69.42
ResNet50	92.24	97.81	97.46	73.86
AlexNet	92.21	98.24	97.88	76.90
Proposed HemNet	94.21	97.46	97.19	76.87

**Figure 6:**
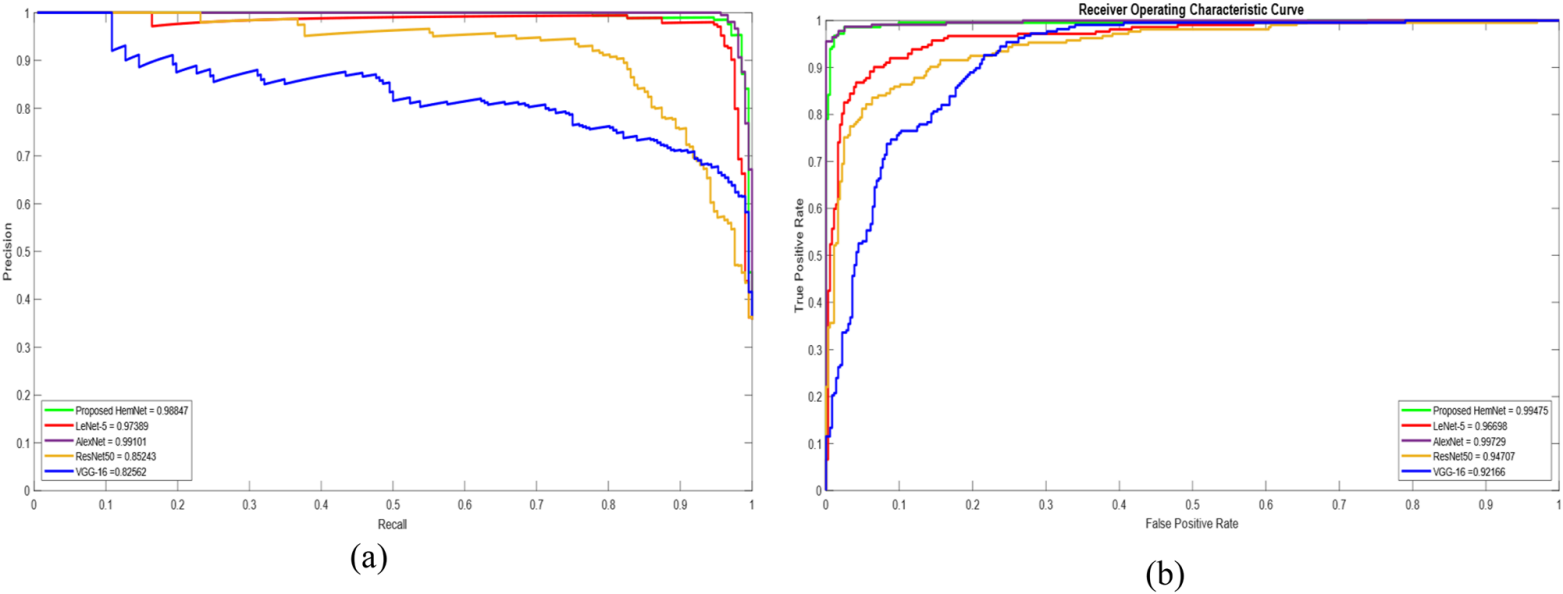
Comparison of Deep Models on the DIARETDB1 Dataset (**a**) PR curves (**b**) ROC curves.

A SoftMax layer of a deep model normalizes each training data example into a probability distribution as a prediction score. These predicted probabilities can be distributed into regions by a threshold classification rule. The precision and recall values change with respect to threshold values. Therefore, it is critical to identify the optimum threshold. Using this evaluation criterion, the best classification model is considered to have a maximum area under the curve (AUC). The precision-recall (PR) curve plots the behavior of a deep model’s precision and recall over threshold values ranging from 0.0 to 1.0. Figure 6a shows the responses of LeNet-5, AlexNet, ResNet50, VGG-16, and proposed HemNet on the DIARETDB1 dataset. The AUC of the proposed HemNet was 0.988, closest to the optimum AUC of 0.991 provided by AlexNet. The plot shows the worst performance of VGG-16 with AUC of 0.826. Similarly, AlexNet and HemNet yielded the highest AUC in the receiver operating characteristic (ROC) curves of deep models can be depicted in Fig. 6b.

## Discussion

**Figure 7:**
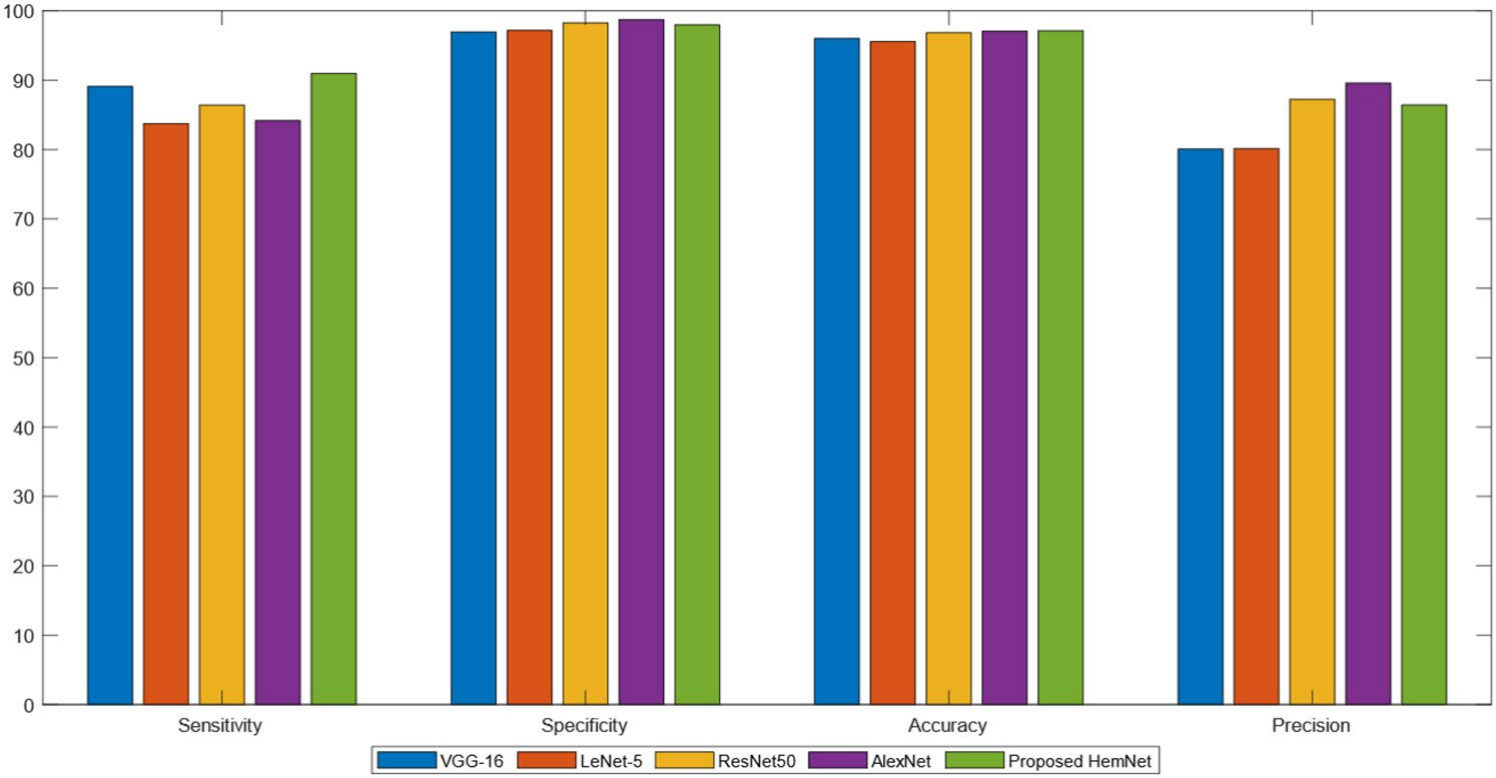
Comparison of Deep Models with Proposed HemNet Network on DIARETDB0 Dataset.

The outcomes of the proposed HemNet model are also encouraging when independently benchmarked on the DIARETDB0 dataset. Effectively, the HemNet marked the highest SE of 90.98% among all the deep networks. However, its SP was less than AlexNet and ResNet50 but higher than VGG-16 and LeNet-5. AC of HemNet was 97.12%, which was also the highest and closer to AlexNet’s 97.08%. Conversely, ResNet50 and AlexNet outclassed the proposed network by scoring the P of 87.25% and 89.58%, respectively. While HemNet marked 86.43% P, which was greater than LeNet-5 and VGG-16. The classification results of various deep learning models on DIARETDB0 are statistically compared in Table 3 and pictorially depicted in Fig. 7.

**Table 3:** Classification of HEs using various models on the DIARETDB0 dataset.

Methods	SE (%)	SP (%)	AC (%)	*P* (%)
VGG-16	89.10	96.95	96.01	80.07
LeNet-5	83.72	97.18	95.57	80.15
ResNet50	86.41	98.27	96.84	87.25
AlexNet	84.17	98.74	97.08	89.58
Proposed HemNet	90.98	97.98	97.12	86.43

The assessment of training time with classification accuracy is problematic because it is laborious due to complex HEs detection applications and cannot be justified. Therefore, this criterion was analyzed using synthetic images by freezing parameters like learning rate and epochs in training. Table 4 explains the results of various deep networks on the concrete crack dataset. Ten epochs were fixed so that the convergence rate could also be assessed. It can be seen from the statistics, the propounded network’s training time was the lowest, which is 11.19 min, but validation and testing accuracies were lower than AlexNet, ResNet50, and VGG-16. The convergence rate of VGG-16 was the highest as it scored 99.70% and 99.50% validation and testing accuracies within the ten epochs. VGG-16 outperformed all other deep models in terms of validation and testing accuracies. However, it took 16.56 min which was more than the 11.19 min of HemNet. Although, the differences in accuracies were not substantial because they could be increased by slightly increasing the epochs.

**Table 4:** Analysis of deep models using time versus classification accuracy on concrete crack dataset.

Method	Time (min)	Validation accuracy (%)	Testing accuracy (%)
LeNet-5	32.50	86.04	86.84
AlexNet	22.15	98.76	98.44
ResNet50	30.57	98.56	98.34
VGG-16	16.56	99.70	99.50
Proposed HemNet	11.19	96.28	95.92

Table 5 elaborates the performances of deep models using the MNIST dataset. The training time of propounded HemNet was 2.33 min which was the minimum, and it scored 99.70 validation and testing accuracies. VGG-16 marked 100% validation accuracy and 99.90 testing accuracy, slightly higher than HemNet. However, its training time was 6.59 min, more than 2.33 of HemNet. The performance metrics of deep models can be observed in Table 5.

**Table 5:** Analysis of deep models using time versus classification accuracy on MNIST dataset.

Method	Time (min)	Validation accuracy (%)	Testing accuracy (%)
LeNet-5	2.53	92.80	91.90
AlexNet	3.11	99.10	98.60
ResNet50	9.52	99.70	99.60
VGG-16	6.59	100	99.90
Proposed HemNet	2.33	99.70	99.70

## Conclusion

The statistics of health organizations from various regions of the world indicate the proliferation of Diabetic retinopathy patients in the future. The insufficient medical resources and time-consuming treatment modalities would be unable to manage the outbreak. Furthermore, the ophthalmologist’s involvement in the screening process and manual inference of the pathology causes adverse effects on the eye due to human error. It demands computer-based algorithms to expedite the screening process and to prevent the side effects of human interpretability. Additionally, these methods can assist in automating the diagnosis process, making it more efficient and less reliant on human interpretation.

This research has demonstrated automatic hemorrhage detection for screening Diabetic retinopathy using a novel hemorrhage network. The detection process is intelligent because it first estimates the prospective hemorrhage’s locations which are imperative to infer the severity level of the ailment. The estimation process generates the data that suffices to deal with the limited data for training a deep model. The propounded network provides promising results while reducing training time significantly. A very deep network may not produce good results for some applications, as the experiment suggests that AlexNet and the proposed network are shallow but provide the highest results. Their overall performance is the best among all comparing networks. VGG-16 scored the best results for simple concrete crack and MNIST datasets. Its convergence rate is the highest. Conversely, its results are worse when applied to complex HEs classification problems. The reason might be the oscillation around the optimum solution due to the excess convergence rate. Therefore, it can be deduced that increasing the network’s layer may not guarantee good results rather than increasing the training time. The arrangement of deep layers and the appropriate selection of parameters are crucial for enhancing the network’s metrics.

The rigorous assessment of the propounded method reveals that the proposed detection scheme depends on seed point extraction. The constituent hemorrhage cannot be detected if a seed point is eliminated during the extraction phase. The parameters of the gaussian matched filter are empirically selected and should not be greater than the cross-section of the main artery. However, manual parameter selection may have adverse effects on the detection process. Therefore, the cross-section of the main artery could be automatically estimated for the robust selection of matched filter parameters and is proposed for future considerations. The feed-forward strategy in the architecture of a deep model might be effective for many applications, as represented by HemNet. Further experimentation needs to be conducted to evaluate the effects of feed-forward on deep networks. However, encouraging evaluation metrics of HemNet have been presented that show the efficacy of HemNet for hemorrhage detection. It is intended to conduct extensive experimentation for assessing this concept in the future.

## Data Availability

Publicly available datasets were analyzed in this study. The datasets can be found here: https://www.it.lut.fi/project/imageret/diaretdb1/index.html, https://www.it.lut.fi/project/imageret/diaretdb0/index.html.
